# The Use of a Digital Well-Being App (Stay Strong App) With Indigenous People in Prison: Randomized Controlled Trial

**DOI:** 10.2196/53280

**Published:** 2024-12-06

**Authors:** Elke Perdacher, David Kavanagh, Jeanie Sheffield, Penny Dale, Edward Heffernan

**Affiliations:** 1 Queensland Forensic Mental Health Service Brisbane, Queensland Australia; 2 School of Psychology University of Queensland Brisbane, Queensland Australia; 3 Centre for Children's Health Research and School of Psychology & Counselling Queensland University of Technology Brisbane, Queensland Australia; 4 Forensic Mental Health Group Queensland Centre for Mental Health Research Brisbane, Queensland Australia

**Keywords:** First Nations, Indigenous, digital mental health, e-mental health, mental health, social and emotional well-being, SEWB, prisoner, prison

## Abstract

**Background:**

Indigenous Australians in custody experience much greater rates of poor mental health and well-being than those of the general community, and these problems are not adequately addressed. Digital mental health strategies offer innovative opportunities to address the problems, but little is known about their feasibility in or impact on this population.

**Objective:**

This study aims to conduct a pilot trial evaluating the impact of adding the Stay Strong app to mental health and well-being services for Indigenous women and men in custody. The trial compared immediate and 3-month delayed use of the app by the health service, assessing its effects on well-being, empowerment, and psychological distress at 3 and 6 months after the baseline.

**Methods:**

Indigenous participants were recruited from 3 high-security Australian prisons from January 2017 to September 2019. The outcome measures assessed well-being (Warwick-Edinburgh Mental Wellbeing Scale), empowerment (Growth and Empowerment Measure [GEM]—giving total, 14-item Emotional Empowerment Scale, and 12 Scenarios scores), and psychological distress (Kessler Psychological Distress Scale). Intention-to-treat effects on these outcomes were analyzed using linear mixed models.

**Results:**

Substantial challenges in obtaining ethical and institutional approval for the trial were encountered, as were difficulties in timely recruitment and retention due to staff shortages and the release of participants from prison before follow-up assessments and an inability to follow up with participants after release. A total of 132 prisoners (age: mean 33, SD 8 y) were randomized into either an immediate (n=82) or a delayed treatment (n=52) group. However, only 56 (42.4%) could be assessed at 3 months and 37 (28%) at 6 months, raising questions concerning the representativeness of the results. Linear improvements over time were seen in all outcomes (GEM total: Cohen *d*=0.99; GEM 14-item Emotional Empowerment Scale: Cohen *d*=0.94; GEM 12 Scenarios: Cohen *d*=0.87; Warwick-Edinburgh Mental Wellbeing Scale: Cohen *d*=0.76; Kessler Psychological Distress Scale: Cohen *d*=0.49), but no differential effects for group or the addition of the Stay Strong app were found.

**Conclusions:**

We believe this to be Australia’s first evaluation of a digital mental health app in prison and the first among Indigenous people in custody. While the study demonstrated that the use of a well-being app within a prison was feasible, staff shortages led to delayed recruitment and a consequent low retention, and significant beneficial effects of the app’s use within a forensic mental health service were not seen. Additional staff resources and a longer intervention may be needed to allow a demonstration of satisfactory retention and impact in future research.

**Trial Registration:**

ANZCTR ACTRN12624001261505; https://www.anzctr.org.au/ACTRN12624001261505.aspx

## Introduction

### Background

Aboriginal and Torres Strait Islander people, the First Nations people of Australia, have maintained >60,000 years of continued cultural connection with >500 nations throughout Australia. Aboriginal and Torres Strait Islander people are made up of distinctive cultural groups with independent governance, languages, traditions, lands, and waterways. These languages and traditions determine and preserve, lore, law, family and kinship relationships, spiritual connections, and well-being.

Aboriginal and Torres Strait Islander people have a higher prevalence of mental disorders and hospitalization for self-harm and suicide than the general population [1-4]. Comparably, it has been found that Indigenous people in custody in Australia have rates of mental health and well-being needs that outstrip those from community surveys of Aboriginal and Torres Strait Islander people [5-7]. Aboriginal and Torres Strait Islander people are more likely to be in contact with the criminal justice system and are 14 times more likely to be incarcerated than non-Indigenous Australians [8,9], representing >30% (13,039/42,970) of all people in prison in Australia yet only >3% of the Australian community [9]. This overrepresentation of Indigenous people in prison and the comparative disadvantage in mental health and well-being needs for Indigenous people are consistently seen in colonized Western countries [10]. The combined factors of agencies targeting their services to the majority population, which do not meet the cultural needs of Aboriginal and Torres Strait Islander people, and the high mental health needs of Aboriginal and Torres Strait Islander people in custody serve to create acute problems with the already limited access to evidence-based, culturally safe and responsive interventions.

Priority government targets can be seen in the National Agreement on Closing the Gap and national principles for forensic mental health, both of which focus on the reduction in the incarceration rate of and health inequality experienced by Aboriginal and Torres Strait Islander people in comparison to non-Indigenous Australians [11-14]. The challenge in increasing access for Indigenous people in custody to evidence-based and culturally responsive interventions and services is that there is minimal expansion in resource provision or reduction in mental health needs in relation to expectations. This means that existing prison mental health services should adopt innovative approaches to meet these needs within the landscape of limited resources available for the whole prison population.

The use of digital mental health apps and programs is an innovative avenue for increasing access for people in custody. This mode of delivery is known for its accessibility and cost-effectiveness [15-18]. While the evaluation of digital mental health strategies for non-Indigenous populations is a growing field [19], the evaluation of digital mental health strategies for Indigenous populations is still very limited. The increased use of digital mental health apps and strategies within health and mental health has occurred with the advancement of technologies, modes of communication, and their accessibility to the general population of Australia, all of which are drivers in this fast-paced, burgeoning field. The speed of change in digital mental health provides great hope for bridging the gap between needs and resources [20-22].

One such resource is the Aboriginal and Islander Mental Health Initiative (AIMhi) Stay Strong app (SSA), which was developed by and specifically for Aboriginal and Torres Strait Islander people and provides a structured guide for mental health and well-being interventions. The focus of this app is on the key determinants of social and emotional well-being (SEWB) for Aboriginal and Torres Strait Islander peoples. The app was first adapted from its hard-copy format (AIMhi Stay Strong Care Plan) to an iOS (Apple operating system, Apple Inc) app in 2013 [23] and then, for this project, to an Android (Google LLC) custody version in 2015 [24]. The hard-copy version from which the app was developed has shown effectiveness in improving well-being, life skills, and alcohol dependence among Indigenous clients with chronic mental illness [25]. For the purpose of this project, the use of the SSA was facilitated by Indigenous Mental Health Intervention Program (IMHIP) practitioners and hosted offline on stand-alone tablets. Through the use of the AIMhi SSA, the aim of this project was to mirror the use of digital mental health resources used in the community and begin addressing the needs of Aboriginal and Torres Strait Islander people in custody.

The initial stage of this project demonstrated the acceptability of the SSA as a digital well-being and mental health tool for use by Indigenous people in custody [24]. Acceptability was tested using postuse qualitative interviews with all IMHIP practitioners (10/37, 27%) and all clients who had completed their second follow-up (27/37, 73%) with IMHIP. A thematic analysis of interviews was conducted using the constant comparison method [26]. Both clients and practitioners identified the SSA’s functionality; engaging appeal; cultural appropriateness; and clinical value in goal-setting, insight, and empowerment, supporting the app as a culturally safe digital mental health and well-being tool for Aboriginal and Torres Strait Islander people in custody [24]. We believe this to be the first example in Australia of a digital mental health app being successfully implemented into service use within the prison environment [24]. However, this study does not undertake research into the efficacy of the app for use with Indigenous people in prison.

### Objective

The primary aim of the Stay Strong Custody project was to conduct a randomized controlled trial within Queensland prisons to determine the feasibility and effectiveness of the SSA. The SSA was added to existing services immediately after a baseline assessment or after a 3-month delay, and well-being, empowerment, and psychological distress were assessed at baseline, 3 months, and 6 months. We hoped to demonstrate the feasibility of conducting research on a digital mental health intervention within prisons and predicted that the SSA would improve the outcomes of participants, relative to existing Indigenous people–specific services.

## Methods

### Setting

The research project was conducted through the IMHIP, a SEWB and mental health service for Aboriginal and Torres Strait Islander people in custody. The IMHIP is an Aboriginal and Torres Strait Islander–led and –staffed service comprising 8 mental health practitioners and 1 manager; the service is also supported by consulting non-Indigenous psychiatrists. The IMHIP is run by the state government health care provider (Queensland Health) within 3 high-security Queensland prisons (Brisbane Women’s Correctional Centre, Southern Queensland Correctional Centre, and Woodford Correctional Centre). High security refers to the most common classification of prisoners in Queensland (93% of the prison population as of December 31, 2022) [27], and in some other jurisdictions, it is termed maximum security. Maximum security in Queensland is provided to only 38 prisoners at any time and involves the isolation of prisoners for the safety and security of other prisoners, staff, and the prison.

### Participants

All Aboriginal and Torres Strait Islander people in custody who were engaged with the IMHIP, who had the capacity to consent, and who caused no safety concerns for staff were invited by the IMHIP staff to participate in this study. This invitation involved both a written and verbal explanation of the study and consent forms. Female participants were recruited between January 2017 and September 2019, while male participants were recruited from January to September 2019 upon the IMHIP’s extension of service to male prisoners.

### Ethical Considerations

The project was approved by the Darling Downs Hospital and Health Service Human Ethics and Research Committee (clearance HREC/14/QTDD/65), the Behavioural and Social Sciences Ethical Review Committee at the University of Queensland (clearance 2015000360), and the Queensland Corrective Services Research Committee (no clearance number) and was conducted in line with the protocol as approved by the ethics committees. IMHIP staff recruited clients who had the capacity to consent to their involvement in the project and who were regarded as not presenting any safety concerns. An invitation to participate in the program involved both written and verbal explanations of the study and consent forms. No financial compensation was provided to participants.

### Outcome Measures

#### Overview

The assessment measures were adapted from hard-copy versions to Android apps specifically for use within this project. Each app had a matching visual interface to the SSA and provided practitioners with an immediate interpretation of the measures for use in sessions; HTML practitioner summaries for download after the session; and an Excel (Microsoft Corp) spreadsheet of demographics, items, and total scores for management and research evaluation purposes. The assessment apps forced the completion of each item before allowing progress to the next item to avoid missing item data.

#### “AIMhi Yarning About Mental Health” Version of the Kessler Psychological Distress Scale App

The “AIMhi Yarning about Mental Health” version [28] of the Kessler Psychological Distress Scale (K10) is an adaptation of the K10 [29]. The full and abbreviated versions of K10 are used widely in the assessment of Aboriginal and Torres Strait Islander people within the public mental health system and national government health surveys [30]. The K10 has 10 self-report items that measure levels of distress and severity of psychological symptoms in the month before the interview. Higher scores represent higher psychological distress. The Likert scale, however, is represented by a rising wedge, providing a pictorial sense of the response scale to better support comprehension by participants.

#### Warwick-Edinburgh Mental Wellbeing Scale App

The Warwick-Edinburgh Mental Wellbeing Scale (WWS) [31] is a 14-item self-report questionnaire that measures mental well-being, including subjective well-being and psychological functioning [32]. Possible scores range from 14 to 70, with higher scores reflecting greater well-being. The WWS version used in this study also substituted the Likert scale with a rising wedge. The use of the WWS for this research project was approved by and registered with NHS Scotland.

#### Growth and Empowerment Measure App

The Growth and Empowerment Measure (GEM) aims to measure psychological and social empowerment, as defined by Aboriginal and Torres Strait Islander people, at individual, family, and organizational levels [33]. The GEM measures emotional well-being and outcomes of empowering change that are important to Indigenous people [33]. The GEM comprises a 14-item Emotional Empowerment Scale (potential range 14-70) and 12 Scenarios (potential range 12-84), with higher scores reflecting greater empowerment. The 14-item Emotional Empowerment Scale uses a rising wedge for responses, and the 12 Scenarios uses pictures of a kauri pine at different stages of growth to symbolize increasing empowerment.

### IMHIP Intervention

All participants received IMHIP treatment as usual. After an initial assessment and triage, IMHIP services at the time of this project comprised psychoeducation; psychological treatments (motivational interviewing, relapse prevention, cognitive behavioral therapy, or narrative therapy strategies); skills training; goal-setting; supportive counseling for problematic substance use; cultural support; healing and support in community connection; transition support (in preparation for release to the community); and general case management. All IMHIP sessions were delivered through an Aboriginal and Torres Strait Islander SEWB cultural model of service. Once a person consented to be a client of the IMHIP, they remained open to receiving services until they withdrew consent, were transferred to a prison where the IMHIP was unavailable, or were released into the community.

### The SSA

The SSA is a culturally safe intervention tool developed to enhance the well-being and mental health of Aboriginal and Torres Strait Islander people. For the purpose of this project, the SSA and 4 other apps were adapted or developed to support IMHIP’s treatment planning and intervention with clients. The use of all apps was facilitated by IMHIP practitioners, requiring no prior computer literacy from clients. All digital resources were written as Android stand-alone apps that could work offline and initially hosted on locked-down tablets.

In consultation with practitioners, additional well-being and mental health resources were also loaded onto the tablets for use in sessions (Figure S1 in Multimedia Appendix 1). This was of benefit to the IMHIP staff, as the tablets then provided the in-reach service with portable workstations and a range of electronic resources for use during the visits of the staff to the prisons.

The Android version of the SSA used was adapted for custody from the original iOS 2013 community version. Tool developers have since released (2022) a hybrid community version of the SSA that can be used on both iOS [34] and Android [35] devices. The key differences between the custody and community versions of the SSA are that the custody version has 2 additional steps and does not have the photo and developer’s “research collection” options on the demographic page or the email option on the client summary page. These changes were made to meet the client confidentiality requirements of both the correctional and health agencies.

**Figure 1:**
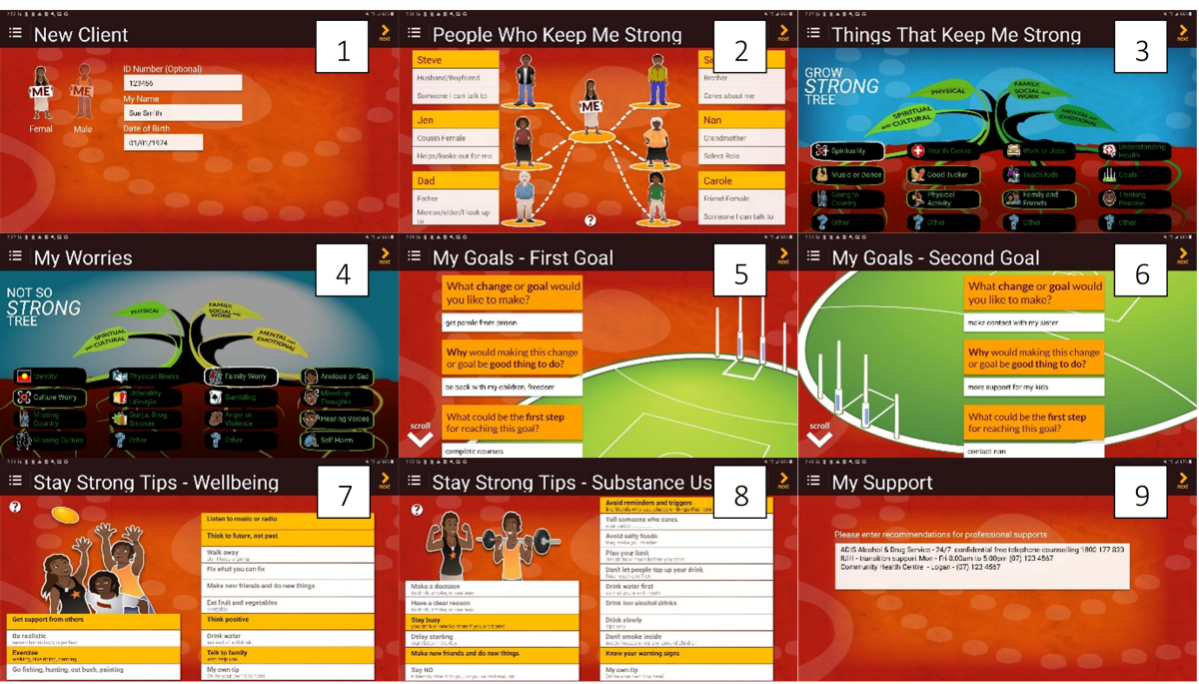
The Stay Strong app custody version steps 1 to 9.

**Figure 2:**
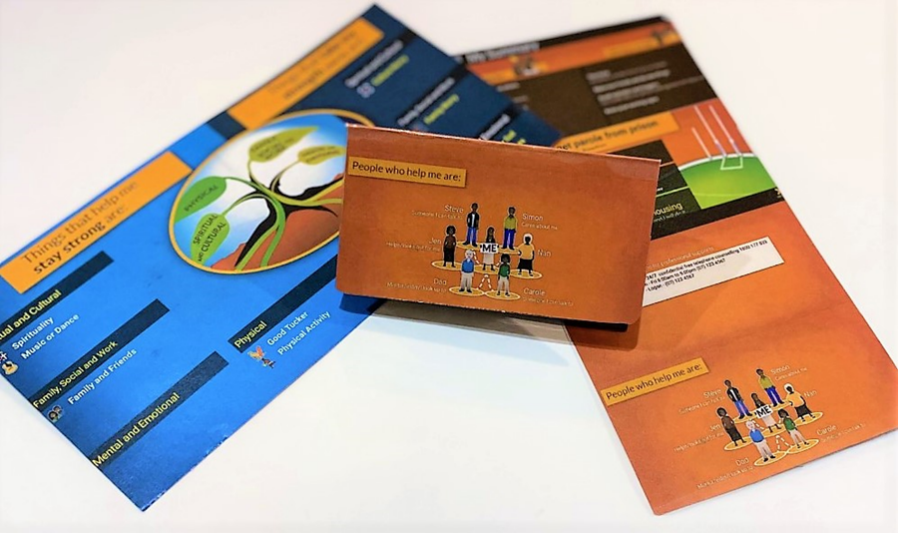
The Stay Strong app custody client card (step 11).

The custody version of the SSA (Figure 1) is an eleven-step intervention involving (1) the collection of demographics, (2) the identification of “people who keep me strong” or support people and the nature of their relationship, (3) the identification of strengths or factors that support client well-being, (4) the identification of worries or factors that reduce client well-being, (5) setting the client’s first goal for change, (6) setting the client’s second goal for change, (7) the provision of well-being tips, (8) the provision of tips to reduce substance use, (9) the provision of “my support” or a description of professional supports and contact details for use upon release from prison, (10) the provision of “client summary” or a review of client information provided across the previous 9 steps, and (11) the provision of a client card to the client (wallet-sized, laminated summary of the SSA; Figure 2).

Items relating to the Stay Strong Tree (Figure 1: steps 3 and 4) provide descriptive data on the strengths and worries that were identified in the SSA sessions. The tree is divided into a four-root system, representative of the four aspects of a client’s life: (1) spiritual and cultural; (2) physical; (3) family, social, and emotional; and (4) mental and emotional aspects. Each root system is then divided into individual strength or worry items (strengths: 16, including 4 open items idiosyncratic to clients; worries: 16 items, including 2 open items idiosyncratic to clients; Figure 1: steps 3 and 4). These items in the SSA represent the key determinants of SEWB for Aboriginal and Torres Strait Islander people.

Clients were provided with 2 copies of their client cards, one for use while in prison and one for their prison property, which would become available to them upon release from prison. Each client card had a summary of the SSA, including professional supports and contact details. The laminated cards were folded in a way that allowed clients to display their support network, or “people who help me are,” in their prison cells (Figure 2).

Outputs of the SSA were the client card, an HTML client summary for reporting purposes, and an Excel data sheet with client responses for management and research evaluation purposes. Unlike the outcome measures, the forced completion of items was not required. The SSA was not written to force completion of items, given the nature of the content, the flexibility required, and the time required to complete this tool. The SSA allows for the completion of steps across more than 1 session. However, all steps in the SSA within this research project were completed in a single session, with practitioners estimating the average time of completion to be 60 minutes.

### Procedure

Following the IMHIP consent and initial intake, clients were identified as potential research participants. IMHIP practitioners then obtained informed consent to participate in the study. All eligible clients provided informed consent. Participants were then randomized into the immediate or delayed intervention group. The generation of the random allocation sequence and the allocation of individual participants were done by the principal researcher (EP), who was not involved in the delivery of the intervention or assessments.

Outcome measures were administered by IMHIP practitioners to all available participants at baseline, 3 months, and 6 months at the beginning of treatment sessions. The immediate intervention group had SSA sessions after each assessment, while the delayed group had sessions only at 3 and 6 months. Each time clients completed or updated an SSA session, they were provided with a summary on a client card (Figure 2). As already mentioned, all (n=27, 100%) the available participants at 6 months also undertook a semistructured feedback interview [24].

### Statistical Analyses

Analyses of the primary outcomes used linear mixed models to allow the presentation of intention-to-treat results. We used *lme4* from RStudio (version 2022.07.2; RStudio, Inc) and incorporated random intercepts for participants in the final models. For the main analyses, we partitioned the effect of time into linear and quadratic contrasts, where the latter captured the departure from linearity that may have resulted from one group obtaining the SSA before the other. For the secondary analyses examining whether the number of strengths and worries changed from the first SSA session (ie, at baseline for the immediate group and 3 months for the delayed group) to the session 3 months later (ie, at 3 months for the immediate group and 6 months for the delayed group). As a result, there were only 2 time points and a single time effect for those analyses.

Because of the small sample size, we restricted the presented model comparisons to ones having (1) only the effects of time; (2) the effects of time, group, and their interaction; and (3) the effects of time, sex, and their interaction. In the interests of a full examination of the results, models with all 3 factors and models with only group or only sex were also examined, but in no case did these models give better fit. In line with CONSORT (Consolidated Standards of Reporting Trials) guidelines, the analyses did not control for any differences between groups at baseline. However, tests for baseline differences are reported in the context of the sample description.

## Results

### Sample Description

A total of 132 participants were recruited: 80 (60.6%) female participants and 52 (39.4%) male participants. The relatively high proportion of female participants (n=80, 60.6%) was reflective of the IMHIP opening their service to male prisoners 2 years after the female IMHIP clients began participating in this study in January 2017. The proportion of female participants to male participants did not differ between intervention groups (*χ*^2^_1_=0.3; *P*=.59). The mean age of participants at baseline was 33 (SD 8.9; range 18-55) years, with no significant difference between groups (*F*_1,130_=0.19, *P*=.66). This age was comparable to the median age of Aboriginal and Torres Strait Islander people in custody in Australia (32.8 years) [9].

Mean scores for the outcome variables in the 2 treatment groups are shown in Table 1. The only difference between the groups that was statistically significant was in the K10 score (*F*_1,119_=4.79, *P*=.03; η^2^=0.039; Table 1). No differences between the scores for men and women approached significance for any outcome.

**Table 1:** Observed means on outcome variables at baseline (N=132).

		Value, n (%)	Value, mean (SD)	Effects for group			
				*F* test (*df*)		*P* value	
**GEM^a^ total**					2.67 (1, 126)		.11
	Delayed	50 (37.9)	96.0 (21.9)				
	Immediate	80 (60.6)	102.6 (24.1)				
	Full sample	130 (98.5)	100.1 (23.4)				
**GEM EES14^b^**					3.29 (1, 125)		.07
	Delayed	49 (37.1)	46.0 (9.1)				
	Immediate	80 (60.6)	49.5 (12.2)				
	Full sample	129 (97.7)	48.2 (11.2)				
**GEM 12S^c^**					1.56 (1, 125)		.21
	Delayed	49 (37.1)	50.0 (14.6)				
	Immediate	80 (60.6)	53.2 (14.1)				
	Full sample	129 (97.7)	52.0 (14.4)				
**WWS^d^**					2.90 (1, 118)		.09
	Delayed	47 (35.6)	41.5 (8.3)				
	Immediate	75 (56.8)	44.7 (10.5)				
	Full sample	122 (92.4)	43.4 (9.8)				
**K10^e^**					4.79 (1, 119)		.03
	Delayed	48 (36.4)	29.3 (6.4)				
	Immediate	75 (56.8)	26.2 (8.9)				
	Full sample	123 (93.2)	27.4 (8.1)				

^a^GEM: Growth and Empowerment Measure.

^b^EES14: 14-item Emotional Empowerment Scale.

^c^12S: 12 Scenarios.

^d^WWS: Warwick-Edinburgh Mental Well-Being Scale.

^e^K10: Kessler Psychological Distress Scale.

### Attrition

**Figure 3:**
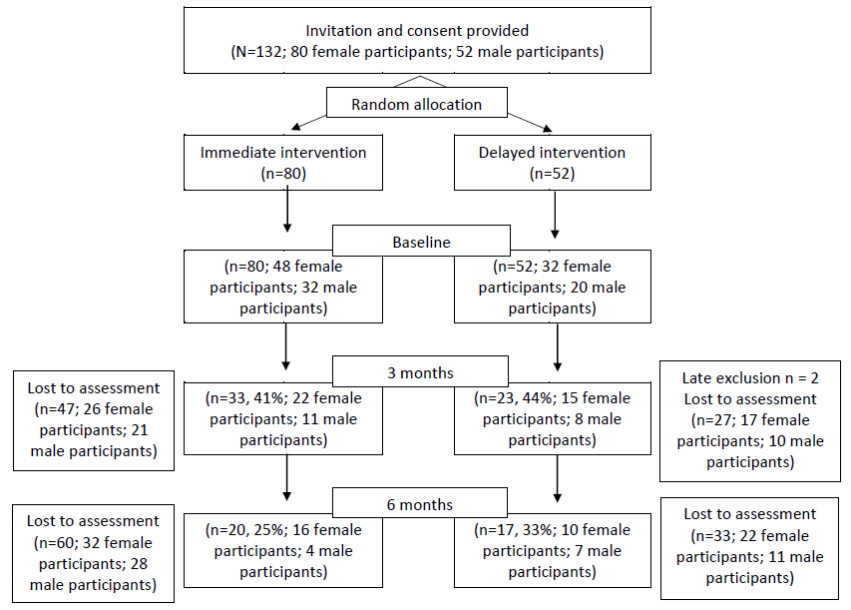
CONSORT (Consolidated Standards of Reporting Trials) diagram.

Among the 54 participants who were originally in the delayed group, 2 (4%) were excluded from the trial because treatment staff considered it imperative for them to receive the immediate intervention. In addition, while the median length of sentence for both female and male clients at the IMHIP was 18 months, many participants had already been imprisoned for some time before recruitment in the study. As a result, a further 74 (%) participants were lost to assessment by the 3-month time point because of release or transfer to another prison. By 6 months, this figure rose to 72% (95/132; immediate: 60/80, 75%; delayed: 35/52, 67%; Figure 3). The CONSORT diagram in Figure 3 represents those participants who completed at least 1 assessment at each time points. Inspection of the figure shows that retention was comparable between the 2 treatment groups.

### SSA Items

The most common strengths and worries that were identified by participants at their first SSA session are shown in Table 2. As may be expected, families or friends were largely identified as both strengths and worries among the Indigenous people in custody.

**Table 2:** The 6 most common strengths and worries at the first Stay Strong app session (n=104).

			Participants, n (%)
**Strength item**			
	Family and friends	95 (91.3)	
	Thinking positive	91 (87.5)	
	Having goals	80 (76.9)	
	Music or dance	75 (72.1)	
	Spirituality	72 (69.2)	
	Physical activity	72 (69.2)	
**Worry item**			
	Family worry	83 (79.8)	
	Anxious or sad	77 (74)	
	Mixed up thoughts	70 (67.3)	
	Own anger or violence	69 (66.3)	
	Gunja (marijuana), grog (alcohol), and smokes (cigarettes)	68 (65.4)	
	Unhealthy lifestyle	54 (51.9)	

Of interest, a comparison was made of participants who fell into the normal (nonsignificant) and severe ranges of psychological distress (K10) at the first time they undertook the SSA (after the baseline assessment for the immediate group and after the 3-month assessment for the delayed group). Those scoring within the normal range of psychological distress had significantly higher levels of social and psychological empowerment (GEM total; *F*_1,58_=30.667, *P*<.001) and well-being (WWS; *F*_1,59_=27.343, *P*<.001) and, according to the SSA items, were more likely to identify work or study (*F*_1,60_=7.558, *P*=.008) as strengthening their well-being. People falling into the severe category of psychological distress identified a significantly greater number of worries (*F*_1,60_=7.098, *P*=.01) and were significantly more likely to identify the following items as negatively impacting their well-being: physical illness (*F*_1,60_=6.668, *P*=.01), their own anger or violence (*F*_1,60_=8.687, *P*=.005), family worry (*F*_1,60_=5.426, *P*=.02), anxiety and sadness (*F*_1,60_=7.882, *P*=.007), and hearing voices (*F*_1,60_=7.622, *P*=.008).

### Primary Outcomes

Despite the substantial loss of participants to posttreatment assessments, results from the linear mixed models are presented in Table 3. As Table 3 shows, adding either group or sex to a model with time alone did not provide a superior fit except for the K10, where a model with group, time, and their interaction gave a slightly better Akaike information criterion and log likelihood. Despite the Bayesian information criterion being slightly higher and the significance of the chi-square falling just short of the .05 level, the more complex model is preferred in this case.

**Table 3:** Comparisons of linear mixed models for main outcomes.

			Comparison model		Parameters, n		AIC^a^		BIC^b^		Log likelihood		Deviance		Chi-square (*df*)		*P* value
**GEM^c^ total**																	
	Group and time	Time only		8		1958.3		1985.4		–971.13		1942.3		3.2 (3)		.36	
	Sex and time	Time only		8		1959.1		1986.3		–971.57		1943.1		2.4 (3)		.50	
	Time only	—^d^		5		1955.5		1972.5		–972.75		1945.5		—		—	
**GEM EES14^e^**																	
	Group and time	Time only		8		1626.7		1653.8		–805.37		1610.7		3 (3)		.39	
	Sex and time	Time only		8		1628.7		1655.8		–806.37		1612,7		1 (3)		.79	
	Time only	—		5		1623.8		1640.7		–806.88		1613.8		—		—	
**GEM 12S^f^**																	
	Group and time	Time only		8		1745.7		1772.8		–864.83		1729.7		2.6 (3)		.45	
	Sex and time	Time only		8		1744.2		1771.3		–864.10		1728.2		4.1 (3)		.25	
	Time only	—		5		1742.3		1759.2		–866.15		1732.3		—		—	
**WWS^g^**																	
	Group and time	Time only		8		1556.6		1583.6		–770.32		1540.6		3.4 (3)		.33	
	Sex and time	Time only		8		1554.9		1581.8		–769.43		1538.9		5.2 (3)		.16	
	Time only	—		5		1554.1		1570.9		–772.04		1544.1		—		—	
**K10^h^**																	
	Group and time	Time only		8		1484.2		1511.2		–734.12		1468.2		7.7 (3)		.05	
	Sex and time	Time only		8		1489.7		1516.7		–736.82		1473.7		2.3 (3)		.52	
	Time only	—		5		1485.9		1502.8		–737.96		1475.9		—		—	

^a^AIC: Akaike information criterion.

^b^BIC: Bayesian information criterion.

^c^GEM: Growth and Empowerment Measure.

^d^Not applicable.

^e^EES14: 14-item Emotional Empowerment Scale.

^f^12S: 12 Scenarios.

^g^WWS: Warwick-Edinburgh Mental Well-Being Scale.

^h^K10: Kessler Psychological Distress Scale.

Parameter estimates for the preferred models are shown in Table 4, and the predicted means are shown in Table 5. For all outcomes, the only significant effect was a linear improvement over time from baseline to 6 months. This is the case even for the K10 scores, where in any case, the greater absolute improvement in means over the study period is seen in the delayed group (due to the nonsignificantly higher absolute mean value at baseline). Effect sizes over the 6 months using Cohen *d* were moderate to large for all variables, with the exception of changes in the K10 scores for the immediate group, which had a Cohen *d* of 0.32 (small; Table 4). Across the whole sample, the effect size for changes in the K10 scores was Cohen *d*=0.49 (moderate).

**Table 4:** Parameter estimates for the preferred model of each outcome.

				Estimate (SE)		*t* test *(df)*		*P* value
**GEM^a^ total**								
	**Intercept**			111.913 (1.989)		56.26 (142.34)		<.001
	**Time**							
		Linear	16.355 (2.245)		7.29 (106.02)		<.001	
		Quadratic	–1.701 (2.118)		–0.80 (87.91)		.42	
**GEM EES14^b^**								
	**Intercept**			53.600 (0.964)		55.60 (145.83)		<.001
	**Time**							
		Linear	7.512 (1.059)		7.10 (107.46)		<.001	
		Quadratic	–0.775 (0.996)		–0.78 (90.74)		.44	
**GEM 12S^c^**								
	**Intercept**			58.355 (1.199)		48.65 (125.52)		<.001
	**Time**							
		Linear	8.874 (1.485)		5.98 (109.87)		<.001	
		Quadratic	–0.936 (1.416)		–0.66 (85.87)		.51	
**WWS^d^**								
	**Intercept**			46.922 (0.845)		55.56 (119.48)		<.001
	**Time**							
		Linear	5.225 (1.052)		4.97 (118.73)		<.001	
		Quadratic	0.084 (1.005)		0.08 ( 94.73)		.93	
**K10^e^**								
	**Intercept**			26.421 (1.148)		23.03 (124.13)		<.001
	**Group**			–2.203 (1.495)		–1.47 (130.87)		.14
	**Time**							
		Linear	–4.322 (1.188)		–3.64 (106.17)		<.001	
		Quadratic	–0.453 (1.144)		–0.40 (90.88)		.69	
	**Group×time**							
		Linear	2.469 (1.601)		1.54 (106.88)		.13	
		Quadratic	1.903 (1.509)		1.26 (91.45)		.21	

^a^GEM: Growth and Empowerment Measure.

^b^EES14: 14-item Emotional Empowerment Scale.

^c^12S: 12 Scenarios.

^d^WWS: Warwick-Edinburgh Mental Wellbeing Scale.

^e^K10: Kessler Psychological Distress Scale.

**Table 5:** Predicted means and effect sizes for each outcome, using the preferred model.

				Values, mean (SD)		SE		*df*		95% CI		Cohen *d* for change from baseline^a^
**GEM^b^ total**												
	Baseline		99.7 (23.6)		1.92		172		95.9-103.0		—^c^	
	3 months		113.3 (18.0)		2.74		216		107.9-119.0		0.56	
	6 months		122.8 (19.4)		3.22		199		116.4-129.0		0.99	
**GEM EES14^d^**												
	Baseline		48.0 (11.3)		0.93		168		46.1-49.8		—	
	3 months		54.2 (9.1)		1.31		215		51.6-56.8		0.55	
	6 months		58.6 (9.2)		1.53		199		55.6-61.6		0.94	
**GEM 12S^e^**												
	Baseline		51.7 (14.5)		1.17		183		49.4-54.0		—	
	3 months		59.1 (11.5)		1.73		215		55.7-62.5		0.52	
	6 months		64.2 (11.6)		2.06		200		60.2-68.3		0.87	
**WWS^f^**												
	Baseline		43.3 (9.8)		0.85		180		41.6-44.9		—	
	3 months		46.9 (8.3)		1.22		211		44.5-49.3		0.37	
	6 months		50.7 (9.2)		1.45		201		47.8-53.5		0.76	
**K10^g^**												
	**Immediate**											
		Baseline	26.1 (8.9)		0.93		161		24.3-28.0		—	
		3 months	23.0 (7.2)		1.28		210		20.5-25.6		0.38	
		6 months	23.5 (8.4)		1.55		193		20.4-26.6		0.32	
	**Delayed**											
		Baseline	29.3 (6.4)		1.16		161		27.0-31.6		—	
		3 months	26.8 (7.5)		1.54		210		23.8-29.8		0.31	
		6 months	23.2 (7.3)		1.73		205		19.8-26.6		0.75	

^a^Calculation of Cohen *d* uses the SD of the full available sample at baseline.

^b^GEM: Growth and Empowerment Measure.

^c^Not applicable.

^d^EES14: 14-item Emotional Empowerment Scale.

^e^12S: 12 Scenarios.

^f^WWS: Warwick-Edinburgh Mental Well-Being Scale.

^g^K10: Kessler Psychological Distress Scale.

### Changes in Strengths and Worries From Baseline to After Treatment

The results are fully displayed in Tables S1-S3 in Multimedia Appendix 1. A model with only time was preferred for strengths, and there was a marginal improvement from the first SSA session to the session 3 months later (t_53.96_=2.00, *P*=.05). For worries, while the model including both time and sex was preferred, this reflected the fact that women had more worries across the study (t_105.73_=–2.25, *P*=.03). Neither time nor time×sex predictors were significantly greater than 0 (t_53.45_=–1.56, *P*=.13 and t_54.17_=0.57, *P*=.57, respectively).

## Discussion

### Efficacy of the SSA

While well-being, empowerment, and psychological distress improved over time for participants, there is no evidence that this could be attributed to the addition of the SSA to IMHIP services. By 6 months, participants’ means for the outcome measures reached those of the validation sample of the GEM (empowerment) and the general Australian population for well-being (WWS) and psychological distress (K10) [33,36,37].

The SSA is a SEWB, culturally informed assessment and brief intervention tool, and given that it was delivered by Indigenous mental health practitioners within a culturally safe SEWB service, it is possible that the SSA could make only minimal potential difference in client outcomes. In addition to the overlap in the process components, there is an overlap in the content of the SSA and core components of the IMHIP model of service that can be seen across assessment, intervention, and transition support. This is particularly evident in the IMHIP’s work with clients using psychotherapeutic assessment and intervention strategies; psychoeducation; skills training; goal-setting; supportive counseling for substance use; cultural support, healing, and community connection; and transition support from prison to the general community.

Even with this said, it is worth noting the positive feedback from both clients and practitioners on the use of the SSA within the IMHIP service [24]. This feedback is highly consistent with the principal researcher’s own experience as a non-Indigenous practitioner, where significant benefits in structuring intakes, assessment, client conceptualization, and treatment appeared to have occurred through the use of the SSA. Greater benefit from the SSA may be seen in services staffed by non-Indigenous practitioners within “mainstream” mental health services than in services staffed by Indigenous practitioners, who may routinely use some elements in their everyday work.

Another potential explanation may be that the SSA was insufficiently intensive or too broad in scope to have an added effect on those participants who remained in the study. While the baseline sample was reflective of the broader incarcerated Indigenous population, participants remaining in prison for 6 months after the baseline may have been a particularly vulnerable group with more complex needs. It remains to be seen whether participants who are released earlier are more suited to a brief intervention than those with longer sentences. Extended follow-up with all participants after their release would allow a test of this possibility as well as an assessment of generalization to their natural environment.

### Secondary Findings

Participants scoring higher on levels of psychological distress (K10) tended to score lower on well-being (WWS) and empowerment (GEM). The WWS, however, does not just provide an indication of the absence of mental illness but rather the presence of mental well-being. Higher scores on this measure signaled enhanced resilience and the presence of protective factors or strengths supportive of the psychological functioning and subjective well-being of participants [31]. Similar to the WWS, the GEM also focuses on the positive aspects of a person’s state. The GEM was used to examine participants’ level of empowerment in their lives. Empowerment is a person’s perception that they have control of their life at a personal and social level that is congruent with their own values [33,38,39].

The participants in this study who presented with higher levels of well-being (WWS) also presented with higher levels of empowerment and lower levels of psychological distress. This is not surprising given the link among mental health, well-being, and empowerment. By removing barriers to clients’ health self-efficacy, for example, through the use of a SEWB health framework in service delivery, we may be able to empower people to actively engage in their recovery and positively impact their well-being and mental health [40-43].

Across the study, an increase in the number of participants’ reported strengths was seen, but worries continued to be at similar levels, with women reporting a higher number of worries. One of the factors affecting progress in participants’ worries may be the limiting factor of being accommodated within a prison. This could be attributed to the added difficulty of trying to address worries from within a structured and closed institutional environment. The most common worry for participants was “family worry,” which IMHIP practitioners identified as typically involving concern over children and family members who were dependent upon the participant for protection or support before the participant’s incarceration. The separation of Aboriginal and Torres Strait Islander people from their families through incarceration was the trigger for this concern, underscoring the strong cultural and kinship obligations and, therefore, the limiting factor to effectively addressing the issue.

Participants scoring higher on psychological distress (K10) were significantly more likely to experience a higher number of worries and worries concerning their family and mental health. Participants’ mental health concerns focused on their own anxiety, depression, and hallucinations. Given that the K10 comprises questions about anxiety and depressive symptoms, it would be expected that people who identified these as concerns in their SSA would also identify them in their responses to the K10. The subclinical or clinical level of participants’ anxiety and depression identified within the SSA was not specified; however, the experience of these conditions and hallucinations would trigger a shared care model between IMHIP and other services. Even with this shared care model, the effective treatment of prisoners’ symptoms of mental illness is still limited by the prison environment, system, resourcing, and culture [44,45]. The limitations placed upon effective intervention by being in prison could, therefore, provide an explanation for the maintenance of participants’ worries. Another explanation for the continued presence of these worries could be that people who were serving longer sentences and were, therefore, available at 6 months may have experienced a higher level of need or issues not easily resolved within the time of the study.

For Aboriginal and Torres Strait Islander people, strengths in cultural identity, connection to culture, and involvement in cultural practices are key determinants of positive SEWB [46,47]. Of the current sample, approximately 69.2% (72/104) identified spirituality and culture as positively affecting their mental health and well-being, demonstrating the importance of having practitioners with expertise in understanding the cultural context of client distress and the capacity to develop pathways for healing and intervention. This has typically been a gap in service delivery for western mental health models of treatment and a key intervention target for the IMHIP. This alternative way of working, weaving cultural expertise and culturally appropriate practices and tools through the SEWB framework, is aimed at improving the well-being and mental health outcomes of people in prison. One of these tools is the SSA, for which, in a published qualitative study, participants of this research project provided feedback, identifying it as culturally appropriate; engaging; and useful in enhancing client empowerment, self-insight, and goal-setting [24].

### Feasibility of Research Approach

The current pilot demonstrated the feasibility of the recruitment, randomization, assessment processes and the implementation of a digital mental health app to the prison environment with Aboriginal and Torres Strait Islander people. This study was, however, not able to demonstrate adequate retention of participants. This difficulty arose due to 2 key factors: the staff to client ratio and the nature of participants’ judicial status.

One-third of the Australian Indigenous prison population is composed of unsentenced people, whose median time spent in prison is 3.4 months, and for sentenced people, the median time spent in prison is 2 years [9]. It was expected that IMHIP clients, being recruited from prisons whose populations were majority sentenced, would have periods of incarceration closer to 2 years. As expected, the median length of sentence for IMHIP clients, both female and male clients, was 18 months. However, the median number of days from allocation to the IMHIP to loss to service (release from prison or transfer to another prison) was 3.3 months. This then resulted in less time between baseline and loss to service, meaning more participants were lost to the evaluation through release from prison or transfer, rather than withdrawal from the study. The overall short-term presence of the IMHIP participants and difficulties in predicting which of the clients would be in custody long enough to complete the evaluation contributed to the marked attrition rate detailed in Figure 3.

In the delay in allocation to the IMHIP and initial assessment, the key case factor was the practitioner to client ratio. At the time of the study, the IMHIP was a new service without permanent funding. This meant that staff were all in temporary positions, adding to the difficulties in recruiting and retaining the staffing profile of an Indigenous-led and Indigenous-staffed SEWB service comprising 8 mental health practitioners. While we see an overrepresentation of Aboriginal and Torres Strait Islander people in prison, we see an underrepresentation of Aboriginal and Torres Strait Islander people in the mental health workforce [48,49]. The IMHIP’s staffing model attempts to begin bridging this gap between client needs and resources, providing a culturally safe, responsive, and innovative service to Aboriginal and Torres Strait Islander people in custody. To address the disparity between the mental health of Indigenous and non-Indigenous people in prison, there needs to be an increase in the number of Indigenous people in the mental health workforce and the use of culturally safe interventions.

Common factors affecting the retention of Indigenous health professionals are salary parity, cultural safety of the workplace, the need for training and specialized supervision, difficulties with overlap between work and community and cultural obligations, the drive to make a positive difference for Indigenous clients, and a shared lived experience with clients; these factors were also linked to emotional fatigue and staff burnout [48,49]. The workforce challenges experienced by IMHIP practitioners fall outside the scope of this paper. These are, however, factors that require further attention, particularly the experience of implementing the IMHIP service delivery model and, more broadly, recommendations to support the implementation of similar workforce models.

### Limitations

While our attempt to conduct a pilot efficacy trial within an existing service (ie, in an effectiveness context) would have been a significant strength if incremental benefits from the SSA were found, it resulted in significant research issues. Staffing limitations within the service led to a high attrition rate, which undermined confidence in the representativeness of the results. This problem may be addressed in future research if sufficient funding is available to ensure rapid recruitment and routine follow-up of participants after release. Limited funding meant that assessments were undertaken by treating staff, who were not blind to allocation and may have introduced response bias. Additional resourcing would enable both single-blind, independent assessments and objective data on the precise nature of usual treatment and the degree of fidelity to the SSA. While the random allocation was independent of the initial assessment and determination of eligibility, it was undertaken by the lead author, and fully automated allocation independent of the research team would improve future studies. A further limitation of this study is that specific data on the mean number of sessions, hours, and types of interventions received by IMHIP clients were not available. A full-scale trial should increase the sample size, which would increase the power of the study to detect any true differences in responses between men and women and identify other predictors of outcome. An increased duration of treatment and follow-up would allow more extensive treatment and an assessment of the extent to which the SSA has sustained effects.

### Conclusions

The adoption of digital mental health services, programs, or apps is considered a pathway to enhancing mental health care and well-being, particularly given their flexibility, accessibility, and cost-effectiveness [50]. The evidence base for digital mental health apps is growing, but the evidence base for the use of for digital mental health apps by prisoners, let alone Indigenous people in prison, is nonexistent.

Evaluations of prison-based, well-being, and mental health interventions for Indigenous people are rare [10] and of digital mental health interventions in prison are nonexistent. This is the first study and evaluation of a prison-based digital mental health app within Australia, to our knowledge, and, certainly, of an Indigenous people–specific digital mental health app. With internet and device access restricted, if not prohibited, in prisons in most countries, this study has become a precedent for the safe adoption and integration of digital mental health initiatives into service provision. We have had an opportunity as early adopters to develop an implementation and efficacy vision for digital mental health in prisons to ensure the design and delivery of digital mental health services that are both clinically and culturally competent.

This research project provided us with an opportunity to rethink our current models of service and the breadth of interventions we can use to support our clients. We began with the development of digital mental health resources and their implementation into our service (in September 2015; Table S4 in Multimedia Appendix 1); then, with the support of stakeholders and clients, we completed a pilot evaluation of a tool, its mode of delivery, and the service through which it was delivered.

The benefits of the SSA, being a culturally validated assessment and intervention tool and delivered by a SEWB service delivery model, are evidenced in the level of engagement and positive feedback of both clients and practitioners [24]. The SSA provides an alternative mode of delivery that can overcome some of the barriers presented by being incarcerated and provide engaging interfaces to assist in improving clients’ readiness or motivation for intervention.

While we were not able to demonstrate a statistically significant benefit for the use of the SSA due to limitations within the study, we were able to demonstrate the identification and resolution of a number of significant administrative and research challenges, and we were able to provide support for further investigation, given the feasibility of the research approach and the positive feedback received from both clients and practitioners [24].

## Appendix

Multimedia Appendix 1 Supplementary tables.

Multimedia Appendix 2 CONSORT (Consolidated Standard of Report Trials) e-Health Checklist (V 1.6.1).

## Abbreviations

AIMhi: Aboriginal and Islander Mental Health Initiative
CONSORT: Consolidated Standards of Reporting Trials
GEM: Growth and Empowerment Measure
IMHIP: Indigenous Mental Health Intervention Program
K10: Kessler Psychological Distress Scale
SEWB: social and emotional well-being
SSA: Stay Strong app
WWS: Warwick-Edinburgh Mental Wellbeing Scale
