# Supplementary material for: The Use of a Digital Well-Being App (Stay Strong App) With Indigenous People in Prison: Randomized Controlled Trial
Source: JMIR Ment Health. 2024 Dec 6;11:e53280. doi: 10.2196/53280 (PMC11662183; doi:10.2196/53280)
Supplement: Multimedia Appendix 1 [file mental_v11i1e53280_app1.docx]

Supplementary Table 1 Comparisons of linear mixed models for strengths and worries

|  | Model | vs. Model | # Parameters | AIC^a^ | BIC^a^ | log Likelihood | Deviance | χ^2^ | df | *p* |
| --- | --- | --- | --- | --- | --- | --- | --- | --- | --- | --- |
|  |  |  |  |  |  |  |  |  |  |  |
| Strengths |  |  |  |  |  |  |  |  |  |  |
|  | 1. Group, Time | 3 | 6 | 726.60 | 744.86 | -357.30 | 714.60 | 1.36 | 2 | .505 |
|  | 2. Sex, Time | 3 | 6 | 724.21 | 742.47 | -356.10 | 712.21 | 3.76 | 2 | .153 |
|  | 3. Time only | .. | 4 | 723.96 | 736.14 | -357.98 | 715.96 | .. | .. | .. |
| Worries |  |  |  |  |  |  |  |  |  |  |
|  | 1. Group, Time | 3 | 6 | 728.04 | 746.31 | -358.02 | 716.04 | 0.50 | 2 | .780 |
|  | 2. Sex, Time | 3 | 6 | 721.93 | 740.19 | -354.97 | 709.93 | 6.61 | 2 | .037 |
|  | 3. Time only | .. | 4 | 724.54 | 736.71 | -358.27 | 716.54 | .. | .. | .. |

^a^AIC: Akaike's Information Criterion; BIC: Bayesian Information Criterion.

Supplementary Table 2 Parameter estimates for the preferred model: strengths and worries

|  | Predictor | Estimate | (SE) | *t* | df | *p* |
| --- | --- | --- | --- | --- | --- | --- |
|  |  |  |  |  |  |  |
| Strengths | (Intercept) | 8.121 | (0.272) | 29.89 | 108.32 | < .001 |
|  | Time | 0.388 | (0.194) | 2.00 | 53.96 | .050 |
|  |  |  |  |  |  |  |
| Worries | (Intercept) | 6.872 | (0. 322) | 21.34 | 102.61 | < .001 |
|  | Sex | -1.165 | (0.518) | -2.25 | 105.73 | .027 |
|  | Time | -0.429 | (0.275) | -1.56 | 53.45 | .125 |
|  | Sex x Time | 0.256 | (0.448) | 0.57 | 54.17 | .571 |
|  |  |  |  |  |  |  |

Supplementary Table 3 Predicted means for strengths and worries, using the preferred model

|  | Mean | (SE) | df | CI |
| --- | --- | --- | --- | --- |
|  |  |  |  |  |
| Strengths |  |  |  |  |
| Pre SSA | 7.85 | (0.27) | 118 | 7.30-7.74 |
| Post SSA | 8.40 | (0.33) | 153 | 7.74-9.05 |
|  |  |  |  |  |
| Worries |  |  |  |  |
| Women |  |  |  |  |
| Pre SSA | 7.17 | (0.33) | 124 | 6.5t2-7.83 |
| Post SSA | 6.57 | (0.42) | 150 | 5.74-7.40 |
| Men |  |  |  |  |
| Pre SSA | 5.83 | (0.41) | 124 | 5.02-6.64 |
| Post SSA | 5.59 | (0.54) | 148 | 4.52-6.65 |

Supplementary Table 4 Steps in implementation of the Stay Strong app in a prison setting [24]

| Steps | Description |
| --- | --- |
| Conception and initiation | Identification of client base, app and context  Scoping potential for project with stakeholders |
| Definition, planning and approval | Development of project plan and evaluation protocol  Gained support from IMHIP, Queensland Forensic Mental Health Service (QH), leadership team of first prison (Brisbane Women’s Correctional Centre, QCS) and Menzies School of Health Research  Gained approval from health (QH) and correctional (QCS) government agencies for implementation of Stay Strong app into service and for evaluation  Gained approval and adapted Stay Strong app to Android custody version  Gained approval and adapted outcome measures from paper versions to Android apps  Development of feedback Android app  Development of locked down tablet interface for project  Practitioners trained in Stay Strong app (incl. Stay Strong app train the trainer) and in use of app in context of IMHIP service  Tested functionality, user interface design, compatibility, performance, installation and security offsite and onsite |
| Launch | September 2015 first IMHIP client to use Stay Strong app (Brisbane Women’s Correctional Centre) |
| Performance and support | Provision of ICT support, clinical supervision, support in output production (practitioner reports, client cards and management data), and continued training for practitioners  Introduction of Android emulator with change to Windows-based tablet PCs |
| Project transition into sustained adoption | Evaluation of Stay Strong app feasibility and efficacy with Indigenous prisoners  Management of transition of technical support from research team and tool developer to health agency  Continued use of Stay Strong app as part of formal IMHIP service delivery model |

Adapted from Perdacher et al, 2022, pg. 4 [24].
